# Impact of Telehealth on Medication Adherence in Chronic Gastrointestinal Diseases

**DOI:** 10.1093/jcag/gwac016

**Published:** 2022-05-14

**Authors:** Hyun Jae Kim, Marcel Tomaszewski, Billy Zhao, Eric Lam, Robert Enns, Brian Bressler, Sarvee Moosavi

**Affiliations:** Division of Gastroenterology, Department of Medicine, University of British Columbia, Vancouver, British Columbia, Canada; Division of Gastroenterology, Department of Medicine, University of British Columbia, Vancouver, British Columbia, Canada; Division of Gastroenterology, Department of Medicine, University of British Columbia, Vancouver, British Columbia, Canada; Division of Gastroenterology, Department of Medicine, University of British Columbia, Vancouver, British Columbia, Canada; Division of Gastroenterology, Department of Medicine, University of British Columbia, Vancouver, British Columbia, Canada; Division of Gastroenterology, Department of Medicine, University of British Columbia, Vancouver, British Columbia, Canada; Division of Gastroenterology, Department of Medicine, University of British Columbia, Vancouver, British Columbia, Canada

**Keywords:** Adherence, Compliance, Gastroenterology, Prescription, Telehealth, Telemedicine

## Abstract

**Background:**

With the COVID-19 pandemic, the demand and availability of telehealth in outpatient care has had exponential growth. Although use of telehealth has been studied and validated for various medical specialties, relatively few studies have looked at its role in gastroenterology.

**Aim:**

To assess effectiveness of telehealth medicine in gastroenterology by comparing medication adherence rate for patients seen with telehealth and traditional in-person appointment for various gastrointestinal conditions.

**Methods:**

Retrospective chart review of patients seen in outpatient gastroenterology clinic was performed to identify patients who were given prescription to fill either through telehealth or in-person appointment. By using provincial pharmacy database, we determined the prescription fill rate.

**Results:**

A total of 206 patients were identified who were provided new prescriptions or prescription renewal at their gastroenterology clinic visit. One hundred and three patients were seen through in-person visit during pre-pandemic period, and 103 patients were seen through telehealth appointment during COVID-19 pandemic. The mean age of patients was 49.2 years (55% female). On average, patients had 4.7 previous visits with their gastroenterologists before their visit. IBD management was the most common reason for visits (37.9% and 46.6% in telehealth and in-person groups, respectively). Prescription fill rate for patients seen through telehealth was 92.2% compared to 81.6% for the in-person group (OR: 2.69, 95% CI: 1.12–6.45; *P* = 0.023).

**Conclusions:**

Medication adherence rate for telehealth visits was higher than for in-patient visits. These findings suggest that telehealth can be an effective method of care delivery, especially for patients with chronic gastrointestinal conditions like IBD.

## Introduction

Telehealth is the use of two-way telecommunication technology to provide health care or information (1). While telehealth has grown exponentially since the beginning of COVID-19 pandemic, interest and application of telehealth was increasing prior to the pandemic (2). There are a number of drivers for its growth. Access to telecommunications technology has improved. It is almost universal that patients have access to a computer and/or cellular device making telehealth readily accessible. Secondly, patients are seeking more convenient and efficient ways to receive their care. This is especially true for access to subspecialized medical care such as gastroenterology. A survey in 2010 conducted on Canadians awaiting gastroenterology consultation found that at least one-third of patients believed their wait time was too long with significant number of patients experiencing impaired quality of life while awaiting consultation because of their gastrointestinal symptoms (3). Expanded use of telehealth can address these issues. Telehealth is reported to significantly reduce wait times for appointments in different medical specialties (4–7). Published economic analyses of telehealth demonstrate cost saving from healthcare system perspective by reducing travel costs, staff wages due to shorter appointments, and reducing hospitalizations (8–10).

While telehealth has application in diverse clinical settings and specialties, it is especially well suited for gastroenterology for a number of reasons. Gastroenterology has significant burden of chronic diseases requiring frequent follow-ups and treatment adjustments in management of patients with inflammatory bowel disease (IBD), gut-brain interactive disorders, and chronic liver disease. Additionally, chronic digestive disease management is arguably symptom and lab driven with comparatively less emphasis on physical examination. Treat-to-target approach of IBD is a clear example of this (11). Unlike management of patients with heart failure which relies heavily on in-person interaction and examinations, management of patients with digestive diseases tends to focus on objective biochemical parameters like fecal calprotectin in addition to clinical symptomatology, while repetitive physical examination is less likely to change the course of the treatment.

Despite its potential, telehealth has had little uptake in gastroenterology prior to the pandemic. According to analysis of data from American Medical Association 2016 survey, nearly a quarter of cardiologists and 15.9% of nephrologists reported using telehealth while only 7.9% of gastroenterologists reported use of telemedicine (12). Gastroenterology was ranked second lowest among internal medicine specialties for telehealth application (13). This is in part due to lack of evidence or research assessing the role of telehealth in gastrointestinal disease management. Compared to other specialties, there is also a disproportionate lack of research in telehealth application in gastroenterology (14). While there have been studies looking at patient and provider satisfaction or perspectives toward telehealth, there have not been enough studies assessing effectiveness of telehealth in objective manner.

The current study is aimed to objectively assess the effectiveness of telehealth in gastroenterology by investigating medication adherence rate when patients are seen through telehealth in comparison to patients seen in-person. Adherence rate was measured by assessing prescription fill rate for patients seen through different visit types when they were given medication prescriptions. Prescription claims data can be an accurate measure of treatment adherence as reported by Dahri *et al.* (15).

## Methods

This is a retrospective cohort study looking at adult patients seen at outpatient tertiary gastroenterology clinic, Pacific Gastroenterology Associates, in Vancouver, B.C. Canada between September 2019 and October 2020. The study was approved by University of British Columbia institutional ethics review board.

### Comparison Group Definition

We identified adult patients who either had a (a) face-to-face in-person visit with a gastroenterologist between September 2019 and March 2020 or a (b) telehealth visit between March 2020 and October 2020. Due to the COVID-19 pandemic impacting outpatient care delivery starting in early 2020, most of outpatient care was provided through telehealth since March 2020, irrespective of patient preference, in order to reduce the spread of COVID-19. Telehealth visits included appointments conducted either through telephone or video conferencing. Two groups were defined based on whether they had in-person or telehealth visit.

By reviewing the clinic’s electronic medical record (EMR), we could determine if a prescription was provided. Patients were eligible for inclusion in the study if they were at least 18 years old and were given a new medication prescription or renewal of their previous prescription during the study period. Patients who were provided with prescription for biologic therapies were excluded from the study. In B.C., the IBD centres have streamlined biologic infusion clinics so that patients can get their biologic therapy without having to personally fill their prescription which makes it difficult to interpret prescription fill data.

### Outcome

The primary outcome of interest was prescription fill rate. This was defined by percentage of prescriptions that was filled within 90 days of being prescribed. We could identify information on prescription fill using the provincial pharmacy database, B.C. PharmaNet (16). The system captures information on all prescriptions dispensed at individual patient level in the province including drug name, dosage, and fill date. It also identifies any prescriptions that were faxed but not filled within 90 days.

### Matching

We matched two groups based on age, gender, number of prior visits with gastroenterologist, providers, proportion of new consultations, and novel medication prescriptions. Due to limited patient size and broad diversity of diseases, two groups were not matched in proportion of diseases. However, they were matched in percentage of patients with IBD and non-IBD.

### Analysis

All statistical analyses were performed using SPSS version 26.0 (SPSS Inc., Chicago, IL). Categorical data were expressed as percentage and continuous data were expressed as mean ± one standard deviation. Power calculation was undertaken based on estimated medication adherence rate of 75% for gastroenterology diseases (17). Based on estimated size of difference of 15%, alpha of 0.05, and beta of 0.80, the required sample size per group is 100 for two-sided t-test. Pearson’s χ^2^ test, fisher’s exact test, or independent samples two-sided T-test were performed to analyze statistical differences in demographics and adherence data between patients who conducted their visit through telehealth or in-person. A univariate analysis was performed with Pearson’s χ^2^ test or independent samples T-test to compare differences in telehealth usage, age, gender, number of prior visits, percentage of new prescription, percentage of new patient to the prescribing gastroenterologist, and percentage of patients with IBD between patients who were adherent versus those who were not. A multivariate analysis was not performed as only one variable was significant on univariate analysis. For all statistical test performed, a *P*-value below 0.05 was considered significant and an odds ratio with 95% confidence interval was reported.

## Results

A total of 206 patients were identified who satisfied the eligibility criteria. Of these, 103 patients were seen by telehealth between March 2020 and October 2020 (telehealth group). One hundred and three patients were seen through in-person visit between September 2019 and March 2020 (in-person group). Demographics and baseline characteristics of study patients are provided in Table 1. Both groups were similar with regard to demographic and patient-specific factors including age, gender, number of previous visits, proportion of new consult visits, and proportion of new medication prescriptions.

**Table 1.** Baseline patient characteristics

Characteristic	Telehealth (*N* = 103)	In-person (*N* = 103)	*P* value
Age—yr	48.8 ± 17.3	49.6 ± 15.6	0.72
Female sex—%	51.5	58.3	0.33
Number of prior visits with gastroenterologist^*^	5.3 ± 6.3	4.1 ± 4.6	0.12
Proportion of new consultations—%^†^	28.2	21.4	0.26
Proportion of novel medication prescription—%^‡^	73.8	74.8	0.87
Proportion of IBD—%^§^	37.9	46.6	0.20

Refers to number of prior appointments patient had with the gastroenterologist seen during index visit.

Proportion of visits that were for patients seen for the first time as an initial consult.

Refers to patients given prescription for medication/s that they were not on at the time of visit.

Proportion of patients seen for IBD-related visit/presentation.

**Figure 1.**
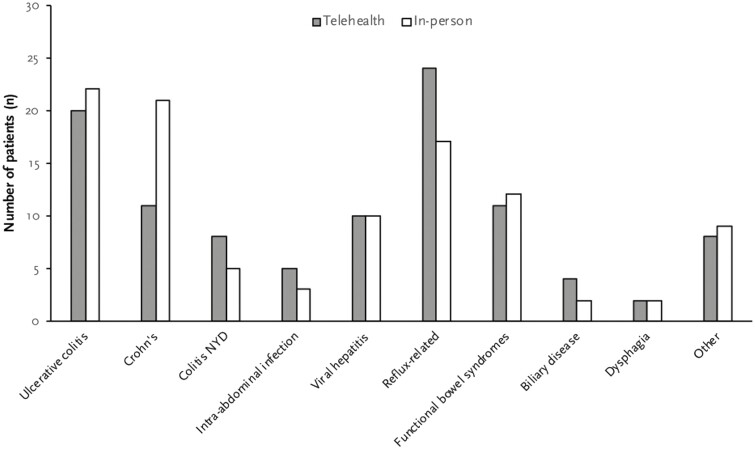
Distribution of diseases for two visit types based on patient’s main presenting symptoms/conditions. 164 × 98 mm (150 × 150 DPI).

We identified ten major disease categories based on patient’s main presenting symptom or disease as illustrated in Figure 1. In both groups, IBD including UC, CD, and undifferentiated IBD was the most commonly managed conditions (37.9% and 46.6% in telehealth and in-person group, respectively, *P* = 0.20). Comparison groups had no significant difference in proportion of IBD-related visits. Other diseases managed included viral hepatitis, intra-abdominal infections, gut-brain interactive disorder, gastroesophageal reflux disease, and pancreaticobiliary disease. Proton pump inhibitors were the most commonly prescribed medication (23.8% of all prescriptions). Ninety-nine out of 103 visits in telehealth group were conducted via audio-only telecommunications while the remaining 4 visits were conducted using video telecommunications.

**Figure 2.**
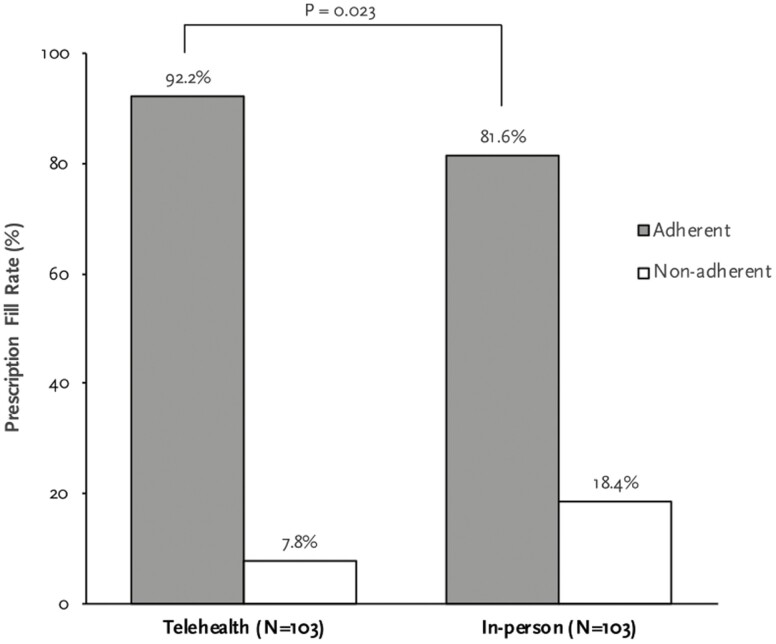
Prescription fill rate compared between telehealth and in-person visits. Prescription fill rate was higher in telehealth group when compared with in-person group. *P* = 0.023, OR: 2.69 (1.12–6.45). 276 × 229 mm (72 × 72 DPI).

The prescription fill rate for telehealth and in-person groups is presented in Figure 2. Prescription fill rate for patients seen through telehealth was 92.2% compared to 81.6% for in-person groups (OR: 2.69, 95% CI: 1.12–6.45; *P* = 0.023). Statistical analysis was performed to identify any factors associated with higher prescription fill rate (Table 2). Only the visit type was found to have significant association with prescription fill rate (OR: 2.69, 95% CI: 1.12–6.45; *P* = 0.023). Notably, age and gender of patients, number of prior visits, new consultation, or novel medication prescriptions had no significant association with the prescription fill rate.

**Table 2.** Univariate analysis of different variables on prescription fill rate

	Adherent	Not adherent	*P* value	OR if significant
% of telehealth visits—%	53.1	29.6	**0.023**	2.69 (CI95%: 1.12–6.45)
Age—yr	49.7 ± 16.7	46.1 ± 15.2	0.29	
% female—%	54.7	55.6	0.94	
Number of prior visits with gastroenterologist	4.9 ± 5.7	3.9 ± 4.1	0.42	
Proportion of novel medication prescription—%	72.1	88.9	0.062	
Proportion of new consultations—%	24.0	29.6	0.53	

Multivariate not completed due to only one variable (visit type) affecting adherence rate. Bolded value of 0.023 was bolded to show that it was statistically significant with *P* value <0.05.

## Discussion

In this retrospective study, we compared prescription fill rate for patients seen through telehealth and in-person visit as a measure of medication adherence. We demonstrated that patients seen through telehealth had significantly higher prescription fill rate compared to patients seen in-person. Our results are in line with previous findings that telehealth is an effective method of providing outpatient medical care (14,18,19). In addition, our findings provide insights into some of commonly held assumptions and concerns regarding telehealth.

It is commonly thought that telehealth is better suited for follow-up visits for patients who already had prior face-to-face visits with their care providers rather than for new consultations (2). In fact, prior to the COVID-19 pandemic telehealth guideline published by American Telemedicine Association suggested requirement for a face-to-face visit prior to a telehealth visit for multiple states (20). In our study’s telehealth group, 28.2% of the patients had first interaction with their gastroenterologists using telehealth. Current findings demonstrated that neither the number of prior visits with the gastroenterologist nor having initial consultation visit via telehealth has a significant impact on the prescription fill rate. While there are certainly situations where telehealth appointment may be less appropriate than face-to-face interaction, telehealth generally appears to be effective with good treatment adherence even when used for initial consultation (21).

Another concern related to telehealth is that the mode of care delivery may be less appropriate for elderly patients presumably given their digital literacy (22). Findings from our study which included patients with wide range of age from 19-year-old to 87-year-old demonstrated no significant association between age of patients and adherence. Although there were insufficient number of elderly patients in our study groups to draw conclusions regarding safety and effectiveness of telehealth for older patients, this may be an important question that future studies could look at.

Previous studies assessing telehealth in gastroenterology had limited generalizability as they relied on enrolling patients who were open to telehealth (23,24). In comparison, all patients in our telehealth group did not have a choice of being seen via telehealth or in-person because the COVID-19 pandemic made in-person visits extremely scarce. Therefore, the study may have included patients who may have had reservations toward telehealth. Additionally, patients in our study had a wide range of gastrointestinal diseases which reflects the depth of patients seen in typical outpatient gastrointestinal practice. This helps further the applicability of telehealth amongst various age group and gastrointestinal diseases. An important strength of our study is that we objectively assessed effectiveness of telehealth by investigating treatment adherence in comparison to traditional face-to-face healthcare visit. This is important as majority of current literature assessing telehealth application are qualitative in nature with lack of objective quality metrics (25–27).

As important as telehealth is, it is important to recognize its limitations. Lack of physical examination means that it has limited utility in assessment of acutely ill or decompensating patients such as acute abdominal pain. It also requires significant initial capital investment as well as training with difficult learning curve (10). There are also limitations to our study. We used prescription fill rate as a measure of adherence. This assumes that filling prescriptions equals to patients using their medications as prescribed which is not necessarily true. Although prescription fill rate from provincial pharmacy database has been used to assess medication adherence, future prospective studies could utilize wider employed methods of assessing adherence such as medication event monitoring system (MEMS) (15,28–30). Two groups of patients in the study were also studied in dramatically different time periods. Telehealth group had visits occurring during the COVID-19 pandemic while in-person group had visits prior to the pandemic. The difference in time period may have been a factor influencing adherence due to patients’ heightened awareness of health or reluctance to physically visit pharmacy to fill their prescription. Another limitation to our study was that we were unable to assess adherence rate of patients receiving biologic therapies. As mentioned before, in B.C. the IBD centres have system in place so that patients can get their biologic infusions in clinic without having to fill their prescription. This unfortunately limited ability to gather or interpret data on this important group of patients. Furthermore, our study did not include potentially important patient and disease variables such as remoteness of patient location, socioeconomic status, experience with telecommunication technology, and disease severity. These may be important factors to consider when using telehealth.

Another concern related to telehealth is that the mode of care delivery may be less appropriate for elderly patients presumably given their digital literacy (22). Although our study results did not demonstrate any significant association between age of patients and adherence, there were insufficient number of older patients in our study groups to draw conclusions regarding use of telehealth for older patients. This is an important question that future studies could assess.

In summary, telehealth is expected to be integral in medical care even after the COVID-19 pandemic is over. With the degree of exposure to telehealth and high levels of satisfaction from both the patients and providers (31), continued presence of telemedicine in healthcare is likely. In order to safely utilize telehealth, we need more information on its impact on clinical outcomes. Our findings demonstrated positive impact of telehealth on treatment adherence rate, but there is still missing information on whether this translates to meaningful clinical outcomes such as lower disease severity, less emergency visits, shortened waitlists, and lower healthcare cost. Further research is required to better understand these aspects of telemedicine.

## References

[1] World Health Organization, ed. Telemedicine: Opportunities and developments in member states: report on the second global survey on ehealth. World Health Organization; 2010.

[2] Perisetti A, Goyal H. Successful distancing: Telemedicine in gastroenterology and hepatology during the COVID-19 pandemic. Dig Dis Sci. 2021;66:945–53. doi:10.1007/s10620-021-06874-x. 33655456 PMC7925138

[3] Paterson W, Barkun A, Hopman W, et al. Wait Times for gastroenterology consultation in Canada: The patients’ perspective. Can J Gastroenterol. 2010;24:28–32. doi:10.1155/2010/912970. 20186353 PMC2830638

[4] Gordon B, Mason B, Smith SLH. Leveraging telehealth for delivery of palliative care to remote communities: A rapid review. J Palliat Care. 2021: 1–13. doi:10.1177/08258597211001184. PMC9286776 33730904

[5] Donnem T, Ervik B, Magnussen K, et al. Bridging the distance: A prospective tele-oncology study in Northern Norway. Support Care Cancer. 2012;20:2097–103. doi:10.1007/s00520-011-1319-1. 22076621

[6] Patel V, Stewart D, Horstman MJ. E-consults: An effective way to decrease clinic wait times in rheumatology. BMC Rheumatol. 2020;4:1–6. doi:10.1186/s41927-020-00152-5. 33073171 PMC7556892

[7] Caffery LJ, Farjian M, Smith AC. Telehealth interventions for reducing waiting lists and waiting times for specialist outpatient services: A scoping review. J Telemed Telecare. 2016;22:504–12. doi:10.1177/1357633X16670495. 27686648

[8] Burns CL, Kularatna S, Ward EC, Hill AJ, Byrnes J, Kenny LM. Cost analysis of a speech pathology synchronous telepractice service for patients with head and neck cancer. Head Neck. 2017;39:2470–80. doi:10.1002/hed.24916. 28963804

[9] Labiris G, Tsitlakidis C, Niakas D. Retrospective economic evaluation of the Hellenic Air Force Teleconsultation Project. J Med Syst. 2005;29:493–500. doi:10.1007/s10916-005-6106-4. 16180485

[10] Snoswell CL, Taylor ML, Caffery LJ. The breakeven point for implementing telehealth. J Telemed Telecare. 2019;25:530–6. doi:10.1177/1357633X19871403. 31631758

[11] Ungaro R, Colombel JF, Lissoos T, Peyrin-Biroulet L. A Treat-to-Target update in ulcerative colitis: A systematic review. Am J Gastroenterol | ACG. 2019;114:874–83. doi:10.14309/ajg.0000000000000183. PMC6553548 30908297

[12] Kane CK, Gillis K. The use of telemedicine by physicians: Still the exception rather than the rule. Health Aff (Millwood). 2018;37:1923–30. doi:10.1377/hlthaff.2018.05077. 30633670

[13] Hepatology TLG. The potential of telemedicine in digestive diseases. The Lancet Gastroenterol Hepatol. 2019;4:185. doi:10.1016/S2468-1253(18)30359-5. 30739659

[14] Borries TM, Dunbar A, Bhukhen A, et al. The impact of telemedicine on patient self-management processes and clinical outcomes for patients with Types I or II Diabetes Mellitus in the United States: A scoping review. Diabetes Metab Syndr. 2019;13:1353–7. doi:10.1016/j.dsx.2019.02.014. 31336491

[15] Dahri K, Shalansky SJ, Jang L, Jung L, Ignaszewski AP, Clark C. Accuracy of a provincial prescription database for assessing medication adherence in heart failure patients. Ann Pharmacother. 2008;42:361–7. doi:10.1345/aph.1K385. 18303147

[16] Health M of. PharmaNet—Province of British Columbia. Accessed May 23, 2020. https://www2.gov.bc.ca/gov/content/health/health-drug-coverage/pharmacare-for-bc-residents/pharmanet

[17] Jackson CA, Clatworthy J, Robinson A, Horne R. Factors associated with non-adherence to oral medication for inflammatory bowel disease: A systematic review. Am J Gastroenterol | ACG. 2010;105:525–39. doi:10.1038/ajg.2009.685. 19997092

[18] Noel K, Messina C, Hou W, Schoenfeld E, Kelly G. Tele-transitions of care (TTOC): A 12-month, randomized controlled trial evaluating the use of Telehealth to achieve triple aim objectives. BMC Fam Pract. 2020;21:1–9. doi:10.1186/s12875-020-1094-5. 32033535 PMC7007639

[19] Castro HK, Cross RK, Finkelstein J. Using a home automated telemanagement (HAT) system: Experiences and perceptions of patients with inflammatory bowel disease. AMIA Annu Symp Proc. 2006;872. PMC1839272 17238492

[20] Lacktman NM, Acosta JN, Levine SJ. 50-State survey of telehealth commercial payer statutes. 2021. <https://www.foley.com/en/insights/publications/2021/02/50-state-telehealth-commercial-insurance-laws> (Accessed November 10, 2021).

[21] Lee T, Kim L. Telemedicine in gastroenterology: A value-added service for patients. Clin Gastroenterol Hepatol. 2020;18:530–3. doi:10.1016/j.cgh.2019.12.005. 31821875

[22] Lam K, Lu AD, Shi Y, Covinsky KE. Assessing telemedicine unreadiness among older adults in the United States during the COVID-19 pandemic. JAMA Intern Med. 2020;180:1389–91. doi:10.1001/jamainternmed.2020.2671. 32744593 PMC7400189

[23] Herrero CP, Bloom DA, Lin CC, et al. Patient satisfaction is equivalent using telemedicine versus office-based follow-up after arthroscopic meniscal surgery: A prospective, randomized controlled trial. J Bone Jt Surg. 2021;103:771–7. doi:10.2106/JBJS.20.01413. 33720907

[24] Cross RK, Arora M, Finkelstein J. Acceptance of telemanagement is high in patients with inflammatory bowel disease. J Clin Gastroenterol. 2006;40:200–8. doi:10.1097/00004836-200603000-00006. 16633120

[25] Integration of Telemedicine Into Clinical Gastroenterology and Hepatology Practice- ClinicalKey. Accessed May 1, 2021. https://www-clinicalkey-com.ezproxy.library.ubc.ca/#!/content/playContent/1-s2.0-S1542356516306681?returnurl=null&referrer=null 10.1016/j.cgh.2016.09.011 27989663

[26] Elkjaer M, Burisch J, Avnstrøm S, Lynge E, Munkholm P. Development of a web-based concept for patients with ulcerative colitis and 5-aminosalicylic acid treatment. Eur J Gastroenterol Hepatol. 2010;22:695–704. doi:10.1097/MEG.0b013e32832e0a18. 19543101

[27] Huang VW, Reich KM, Fedorak RN. Distance management of inflammatory bowel disease: Systematic review and meta-analysis. World J Gastroenterol. 2014;20:829–42. doi:10.3748/wjg.v20.i3.829. 24574756 PMC3921492

[28] Lau HS, de Boer A, Beuning KS, Porsius A. Validation of pharmacy records in drug exposure assessment. J Clin Epidemiol. 1997;50:619–25. doi:10.1016/s0895-4356(97)00040-1. 9180655

[29] Steiner JF, Prochazka AV. The assessment of refill compliance using pharmacy records: Methods, validity, and applications. J Clin Epidemiol. 1997;50:105–16. doi:10.1016/s0895-4356(96)00268-5. 9048695

[30] Hertz RP, Unger AN, Lustik MB. Adherence with pharmacotherapy for type 2 diabetes: A retrospective cohort study of adults with employer-sponsored health insurance. Clin Ther. 2005;27:1064–73. doi:10.1016/j.clinthera.2005.07.009. 16154485

[31] Kruse CS, Krowski N, Rodriguez B, Tran L, Vela J, Brooks M. Telehealth and patient satisfaction: A systematic review and narrative analysis. BMJ Open. 2017;7:1–12. doi:10.1136/bmjopen-2017-016242. PMC5629741 28775188

